# The short-term effectiveness of balance taping on acute nonspecific low-back pain: A case report

**DOI:** 10.1097/MD.0000000000009304

**Published:** 2017-12-22

**Authors:** Jung-Hoon Lee

**Affiliations:** Department of Physical Therapy, College of Nursing and Healthcare Sciences and Human Ecology, Dong-Eui University, Eomgwangno, Busanjin-gu, Busan, Republic of Korea.

**Keywords:** balance taping, contact test, kinesiology tape, low-back pain, movement assessment

## Abstract

**Rationale:**

Low back pain has a significant socioeconomic impact. Repetitive lifting, with combined twisting and flexion motions of the lumbar spine, increases the risk for low-back pain and injury to the supporting tissues.

**Patient concerns:**

A 60-year-old male who presented with acute low-back pain, with a pain intensity of 6/10 on the visual analog scale (VAS) and an Oswestry disability index (ODI) score of 70%. The range of motion (ROM) of the lumbar spine on initial examination, relative to the normal peak ROM, was as follows: extension, 12°/30°; flexion, 15°/80°; left rotation, 15°/45°; and right rotation, 25°/45°.

**Diagnoses:**

He was diagnosed as acute nonspecific low-back pain sustained with repetitive lifting, combining motions of flexion and twisting.

**Interventions:**

The balance taping was applied for 16 h/day, on average, for 3 consecutive days was used as the primary treatment to manage the patient's low-back pain.

**Outcomes:**

The application of balance taping increased the range of motion of the lumbar spine as follows: flexion, from 15° to 77°; extension, from 12° to 27°; right rotation, from 25° to 45°; and left rotation, from 15° to 45°. The ODI score decreased from 70% to 0%, and the VAS score from 6/10 to 0.

**Lessons:**

We propose that balance taping using kinesiology tape could serve as a complementary approach to other treatments for the treatment of acute nonspecific low-back pain.

## 1 Introduction

Low-back pain has a significant socioeconomic impact.^[1]^ Typically, low-back pain results from lifting an unexpected load, which creates a loss of mechanical balance of the lumbosacral spine,^[2]^ including injury to the osteoligamentous tissues that support the lumbar spine against high flexion loads.^[3]^ Repetitive lifting, with combined twisting and flexion motions of the lumbar spine, increases the risk for low-back pain and injury to the supporting tissues.^[4]^ The aim of this case report was to evaluate the effectiveness of balance taping, using kinesiology tape, in reducing acute nonspecific low-back pain due to repeated lifting. The patient provided informed consent for publication of this case report.

## 2 Case report

A 60-year-old male presented with acute nonspecific low-back pain sustained with repetitive lifting, combining motions of flexion, and twisting. The patient reported a pain intensity of 6/10 on the visual analog scale (VAS) and an Oswestry disability index (ODI) score of 70%, where the ODI score can range between 0%, no disability, and 100%, maximum disability. The range of motion (ROM) of the lumbar spine on initial examination, relative to the normal peak ROM,^[5]^ was as follows: extension, 12°/30°; flexion, 15°/80°; left rotation, 15°/45°; and right rotation, 25°/45°.

**Figure 1:**
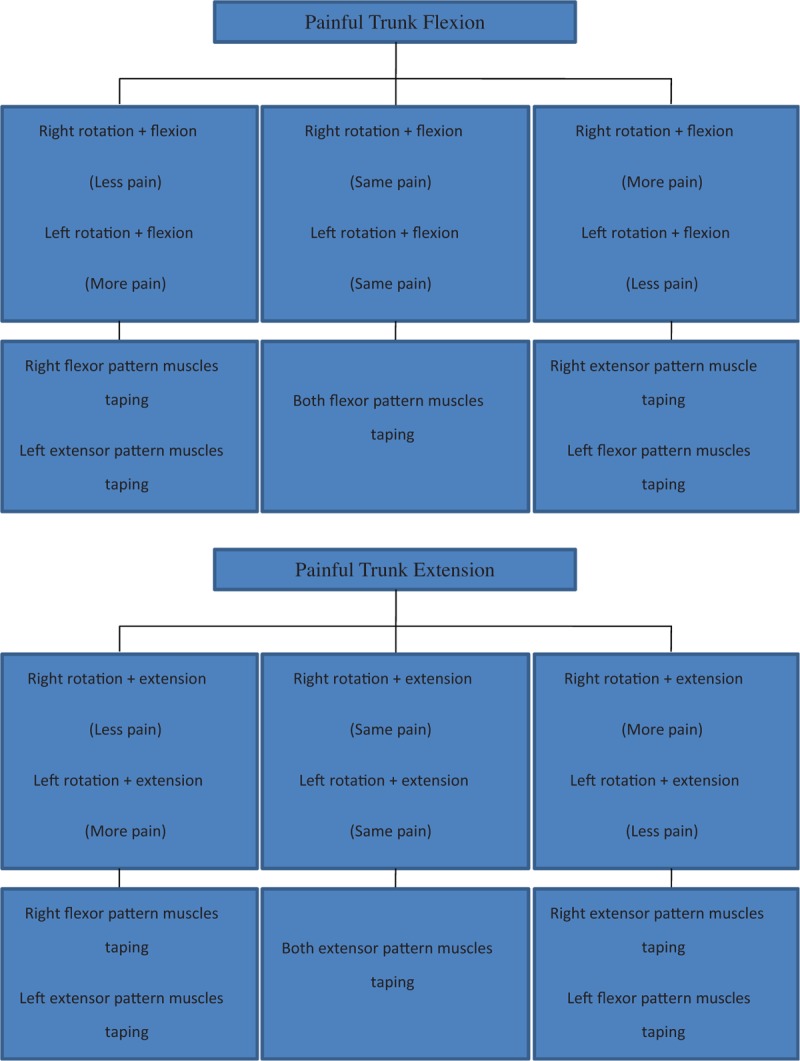
Movement test to identify the pattern of pain (direction, symmetric/asymmetric) for application of the balance taping.

The movement test (shown in Fig. 1) and the contact test were performed to determine the pattern of pain and to identify the muscles over which the taping should be applied, respectively.^[6]^ The aim of the first phase of the movement test is to identify the most painful movement, which for our patient was forward trunk flexion. The second phase of the movement test consists of determining if the pain presents as a symmetric or an asymmetric pattern, with flexion performed in combination with trunk rotation.^[7]^ For our patient, a asymmetric pattern of pain was identified, with greater pain observed with combined flexion and left rotation than with combined flexion and right rotation. Therefore, we considered the left-sided pain to be an extensor pattern and the right-sided pain a flexor pattern.^[7]^ To ascertain the contribution of specific muscle groups to the pain, the contact test was performed (Table 1), with the examiner applying pressure on the skin overlying the muscles identified in the movement test,^[6,7]^ specifically an extensor pattern to the left and a flexor pattern to the right. Pain was reduced with contact pressure on the left side of the trunk, over the internal oblique, quadratus lumborum, lower trapezius, and latissimus dorsi muscles, and on the right side, with contact pressure over the external oblique, upper trapezius and rectus abdominal muscles.

**Table 1:**
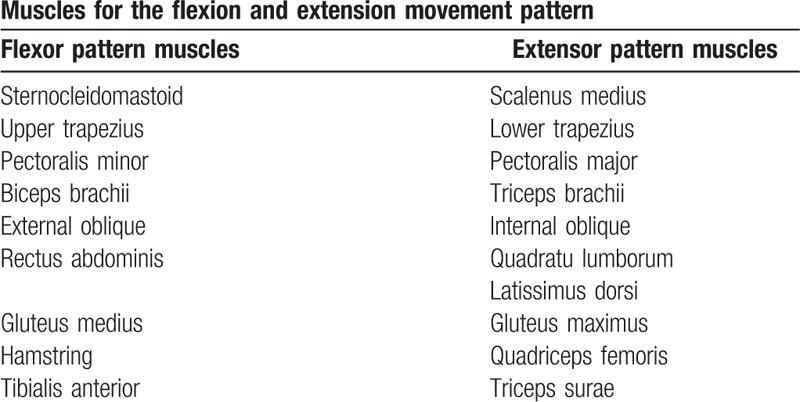
Muscles for the flexion and extension movement pattern.

**Figure 2:**
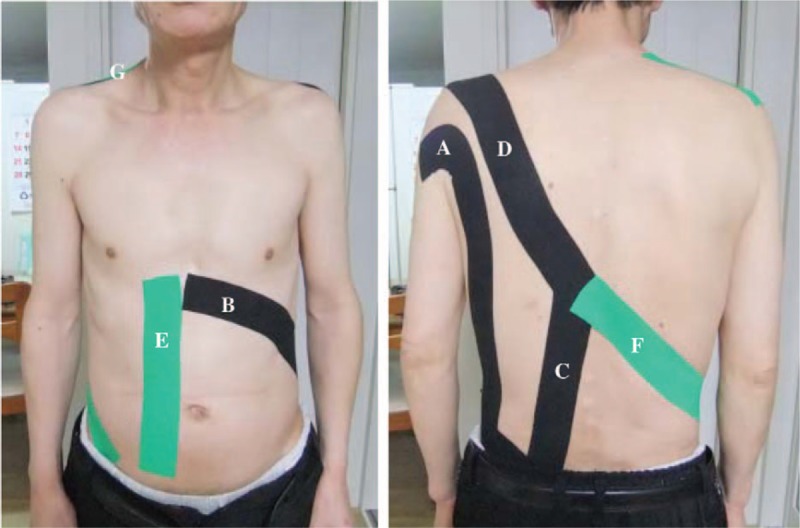
Balance taping used to manage the acute nonspecific low-back pain in this case.

The balance taping was applied for 16 h/day, on average, for 3 consecutive days was used as the primary treatment to manage the patient's low-back pain. The traditional application of kinesiology tape suggested by Kase^[8]^ requires that the target area be placed in a stretched position to apply the tape. In our patient, however, the forward flexion posture required to apply the tape to the low-back-caused pain. Therefore, the balance taping was applied with the patient in a forward leaning position, with hands on a table placed in front of him to provide support. The kinesiology tape (BB TAPE, WETAPE Inc, Paju, Korea) was applied over the muscles identified in the contact test, with 15% to 20% of prestretch on the tape. In order to prevent skin irritation, the ends of kinesiology tape (approximately 2–3 cm) were applied without being stretched.^[6,9–11]^ For the left latissimus dorsi,^[6,12]^ the tape was applied from the spinous process of the sacrum to the lesser tubercle of the humerus (Fig. 2A). For the internal oblique muscle,^[6,12]^ the tape was applied from the middle one-third of the intermediate line of the iliac crest to below the xiphoid process (Fig. 2B). For the quadratus lumborum muscle,^[7]^ the tape was applied from the 12th rib and transverse processes of upper lumbar vertebrae to the iliac crest (Fig. 2C). For the lower trapezius muscle,^[7]^ the tape was applied from the acromion to the spinous process of the 10th thoracic vertebra (Fig. 2D). For the right rectus abdominal muscle,^[6,12]^ the tape was applied from the symphysis pubis to the xiphoid process and the 5^th^ and 6th costal cartilages (Fig. 2E). For the external oblique muscle,^[6,12]^ the tape was applied from the 10th to 11th thoracic vertebrae to below the left anterior superior iliac spine (Fig. 2F). For the upper trapezius muscle,^[6]^ the tape was applied from below the mastoid process to the ipsilateral acromion (Fig. 2G).

Immediately after application of the balance taping, ROM increased to: 20°/30° in extension; 50°/80° in flexion; 40°/45° in left rotation; and 45°/45° in right rotation. Following the third application of the balance taping (day 3), the ODI score decreased from 70% to 0%, and the VAS from 6/10 6 to 0. The decrease in pain further improved the ROM of the lumbar spine to: 27°/30° in extension; 77°/80° in flexion; 43°/45° in left rotation; and 45°/45° in right rotation.

## 3 Discussion

This case study demonstrates the effectiveness of balance taping, applied on 3 consecutive days for 16 h/day, on average, in decreasing acute nonspecific low-back pain and improving ROM of the lumbar spine, supporting the findings of Arikawa Isao.^[7]^ An important component of the assessment is to determine the asymmetric and symmetric distribution of the low-back pain, which may be reflective of the tonic symmetric and asymmetric neck reflexes.^[7]^ Generally, muscle patterns that involve flexion or external rotation of the shoulder and hip are defined as flexor patterns, while muscle patterns that involve extension or internal rotation of the shoulder or hip or trunk rotation are defined as extensor patterns.^[7]^

For this patient, we applied a modified movement test to confirm an asymmetric pattern of pain, with greater pain reported for the combination of trunk flexion and right rotation, indicative of a right flexor pattern and a left extensor pattern.^[7]^ Using the contact test as a follow-up,^[6]^ we identified the following muscles to be contributing to the pain: the left internal oblique, quadratus lumborum, lower trapezius, and latissimus dorsi muscles, and the right external oblique, upper trapezius and rectus abdominal muscles. Application of the tape improved overall ROM, with a decrease in pain on combined flexion and left rotation.

The decrease in pain with the application of the balance taping is likely due to the stimulation of low-threshold cutaneous mechanoreceptors,^[13,14]^ located in the muscles, joints, tendons, and skin.^[6]^ Stimulation of these mechanoreceptors activates large diameter fibers, such as A-beta fibers, which reduces pain transduction along the small nociceptive fibers, namely the C and A-delta fibers, via activation of inhibitory interneurons.^[15]^ As well, the application of balance taping activates gamma motor neurons, indirectly via an attenuation of Ia afferents.^[13]^ This activation of gamma motor neurons has been shown to improve muscle weakness of the quadriceps, with application of kinesiology tape to the knee.^[13]^ Activation of gamma motor neurons may also improve the “smoothness” of the contraction of targeted muscles and improve the muscle coordination for ROM, further lowering pain on movement.

In interpreting our findings, it is important to note that this is a single case study with no comparative analysis to other treatments or taping methods. Based on our experience, we propose that balance taping using kinesiology tape could serve as a complementary approach to other treatments for the treatment of acute nonspecific low-back pain.

## References

[1] Blahova Z Holm JC Weiser T Nicoboxil/nonivamide cream effectively and safely reduces acute nonspecific low back pain—a randomized, placebo-controlled trial. J Pain Res 2016;9:1221–30. 28008281 10.2147/JPR.S118329 PMC5167490

[2] Commissaris DA Toussaint HM Load knowledge affects low back loading and control of balance in lifting tasks. Ergonomics 1997;40:559–75. 9149555 10.1080/001401397188035

[3] Adams MA Dolan P A technique for quantifying bending moment acting on the lumbar spine in vivo. J Biomech 1991;24:117–26. 2037611 10.1016/0021-9290(91)90356-r

[4] Marras WS Lavender SA Leurgans SE The role of dynamic three-dimensional trunk motion in occupationally-related low back disorders. Spine 1993;18:617–28. 8484154 10.1097/00007632-199304000-00015

[5] Clarkson HM Musculoskeletal Assessment: Joint Range of Motion and Muscle Strength. 2nd edn Philadelphia: Lippincott Williams & Wilkins; 2000.

[6] Lee JH Choi SW Balance Taping: Clinical Application of Kinesiology Tape for Musculoskeletal Disorders. Paju: WE TAPE; 2016.

[7] Kim GW Orthopedic medical taping treatment. Goyang: Daeseong Medical Book; 2004.

[8] Kase K Wallis J Kase T Clinical Therapeutic Applications of the Kinesio Taping Method. 4th edn Tokyo: Kinesio Taping Association; 2003.

[9] Han JH Lee JH Yoon CH The mechanical effect of kinesiology tape on rounded shoulder posture in seated male workers: a single blinded randomised controlled pilot study. Physiother Theory Pract 2015;31:120–5. 25264014 10.3109/09593985.2014.960054

[10] Hwang-Bo G Lee JH Kim HD Efficacy of kinesiology taping for recovery of dominant upper back pain in female sedentary worker having a rounded shoulder posture. Technol Health Care 2013;21:607–12. 24225408 10.3233/THC-130753

[11] Kim BJ Lee JH Kim CT Effects of ankle balance taping with kinesiology tape for a patient with chronic ankle instability. J Phys Ther Sci 2015;27:2405–6. 26311206 10.1589/jpts.27.2405 PMC4540890

[12] Hwang-Bo G Lee JH Effects of kinesio taping in a physical therapist with acute low back pain due to patient handling: a case report. Int J Occup Med and Environ Health 2011;24:320–3. 21845524 10.2478/s13382-011-0029-8

[13] Konishi Y Tactile stimulation with kinesiology tape alleviates muscle weakness attributable to attenuation of Ia afferents. J Sci Med Sport 2013;16:45–8. 22682093 10.1016/j.jsams.2012.04.007

[14] Bravi R Quarta EEJ Cohen EJ A little elastic for a better performance: kinesiotaping of the motor effector modulates neural mechanisms for rhythmic movements. Front Syst Neurosci 2014;8:181. 25309355 10.3389/fnsys.2014.00181 PMC4174732

[15] Martini FH Fundamentals of Anatomy and Physiology. 4th edn United States of America: Prentice Hall; 2004.

